# Supplementary material for: LaDIVA: A neurocomputational model providing laryngeal motor control for speech acquisition and production
Source: PLoS Comput Biol. 2022 Jun 23;18(6):e1010159. doi: 10.1371/journal.pcbi.1010159 (PMC9258861; doi:10.1371/journal.pcbi.1010159)
Supplement: S1 Text — Simulation responses of the LaDIVA model for the vocal fo reflexive paradigm plotted over a longer period of time. (DOCX) [file pcbi.1010159.s002.docx]

**S1 Text**

**Detailed calculation of compensatory response magnitude for the vocal *f_o_* reflexive perturbation of +100 cents when full auditory feedback compensation is considered (i.e.,** $\mathbf{g}_{\boldsymbol{fb\_aud}}$**=1; See *Fig 3C* of the manuscript).**

Here we consider a baseline vocal *f_o_* of 134 Hz in the model as presented in Cases A, B, C, and D. Equation SE1 provides the Hz to cents conversion. vocal $f_{value(Hz)}$ is 134 Hz in the model as presented in Cases A, B, C, and D. Equation E1 provides the Hz to cents conversion.

$$\begin{aligned} C \left( \mathrm{cents} \right)=1200*{log}_{2}\left( \frac{f_{baseline(Hz)}+f_{value(Hz)}}{f_{baseline(Hz)}} \right)\#\left( E1 \right) \end{aligned}$$

|  | Hz value | Conversion | Cents value | Calculation |
| --- | --- | --- | --- | --- |
| Baseline Frequency | 134 Hz |  |  |  |
| Perturbation (difference from baseline) | 7.9681 Hz | **←** | +100 cents | $100= 1200*{log}_{2}\left( \frac{134+f_{value(Hz)}}{134} \right)$ |
| Perturbation output (baseline + perturbation) | 141.9681 Hz |  |  | 134 + 7.9681 (Hz) |
| Target region maximum (difference from baseline) | 0.3876 Hz | **←** | 5 cents | $5= 1200*{log}_{2}\left( \frac{134+f_{value(Hz)}}{134} \right)$ |
| Target region maximum (baseline + target max) | 134.3876 Hz |  |  | 134 + 0.3876 (Hz) |
| Total error calculated by model (perturbation output - target max) | 7.5805 Hz | **→** | 95.2675 cents | 141.9681 – 134.3876 (Hz) |
| If 50% of this error is incorporated to final output signal, error =total error/2 | 3.7903 Hz |  |  | 7.5805 Hz / 2 |
| Compensatory signal = (-1)* error | -3.7903 Hz | **→** | -49.6753 cents | $C (cents)= 1200*{log}_{2}\left( \frac{134-3.7903}{134} \right)$ |


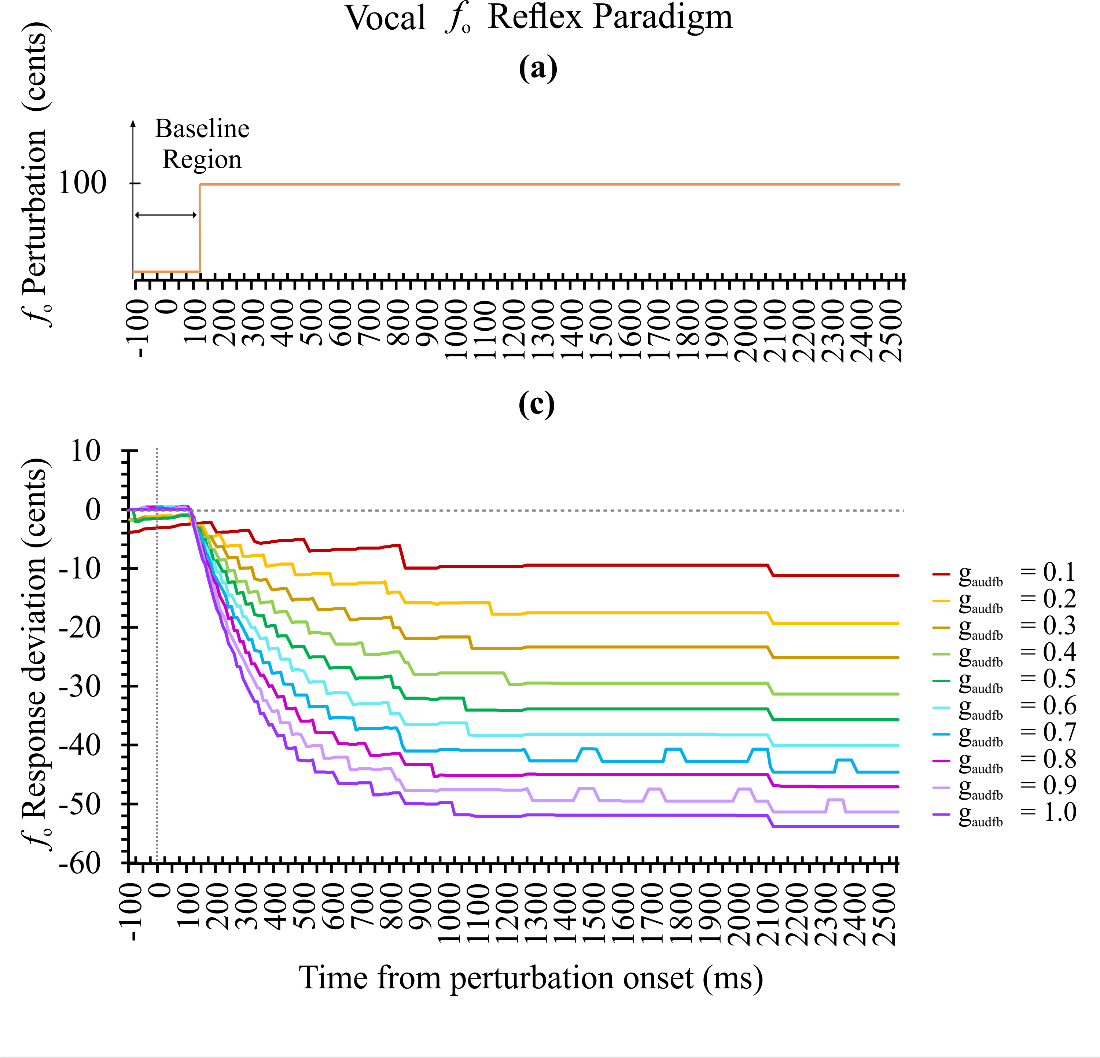


***Fig A: Simulation responses of the LaDIVA model for the vocal f_o_ reflexive paradigm plotted over a longer period of time*.** (a) Applied *f_o_* perturbation for a perturbed trial in the vocal *f_o_* reflexive paradigm. (b) Simulation responses for vocal *f_o_* reflexive paradigm plotted for a 3 second duration. Note that the response plateaus at approximately 50 cents for g_audfb_ =1 when observed over a long period of time.
