# Supplementary material for: LaDIVA: A neurocomputational model providing laryngeal motor control for speech acquisition and production
Source: PLoS Comput Biol. 2022 Jun 23;18(6):e1010159. doi: 10.1371/journal.pcbi.1010159 (PMC9258861; doi:10.1371/journal.pcbi.1010159)
Supplement: S2 Text — (DOCX) [file pcbi.1010159.s003.docx]

**S2 Text**

**Vocal *f_o_* adaptive response when production noise is included in intrinsic laryngeal muscle activation levels (Compared against adaptive paradigms response in Fig 3D of the manuscript).**

Trial-to-trial noise in speech acquisition, production, and learning are inherent characteristics in empirical data related to speech motor control (Cumbers, 2013 [1]; Dhawale et al., 2017 [2]; Scheerer & Jones, 2012 [3]). However, the LaDIVA model does not incorporate production or perception noise in its current implementation. Therefore, the simulation outputs are noise-free and smooth across trials. We incorporated production noise for the motor representations in LaDIVA to mimic production variability. For this, we included a noise source with normal distribution (i.e., N(0,1), mean = 0, variance =1), with a production noise parameter ɛ that was changed between 0.001 – 0.050 to vary the noise level. This noise source was incorporated to the cricothyroid (CT) and thyroarytenoid (TA) muscle activation levels in the model See Equations E1 and E2.

$$a_{CT} = a_{CT}+ \varepsilon*N\left( 0,1 \right) \left( E1 \right)$$

$$a_{TA}= a_{TA}+ \varepsilon*N(0,1) \left( E2 \right)$$

We observed production noise-based variations in the simulated adaptive paradigm outputs when different levels of production noise were included in the LaDIVA model. Adding noise-based variability at production, perception, and/or learning levels will be carried out in future iterations as it requires qualitative and quantitative specification of noise characteristics (i.e., ɛ as well as the mean and variance of the noise distributions for production and perception) via behavioral paradigms.

**
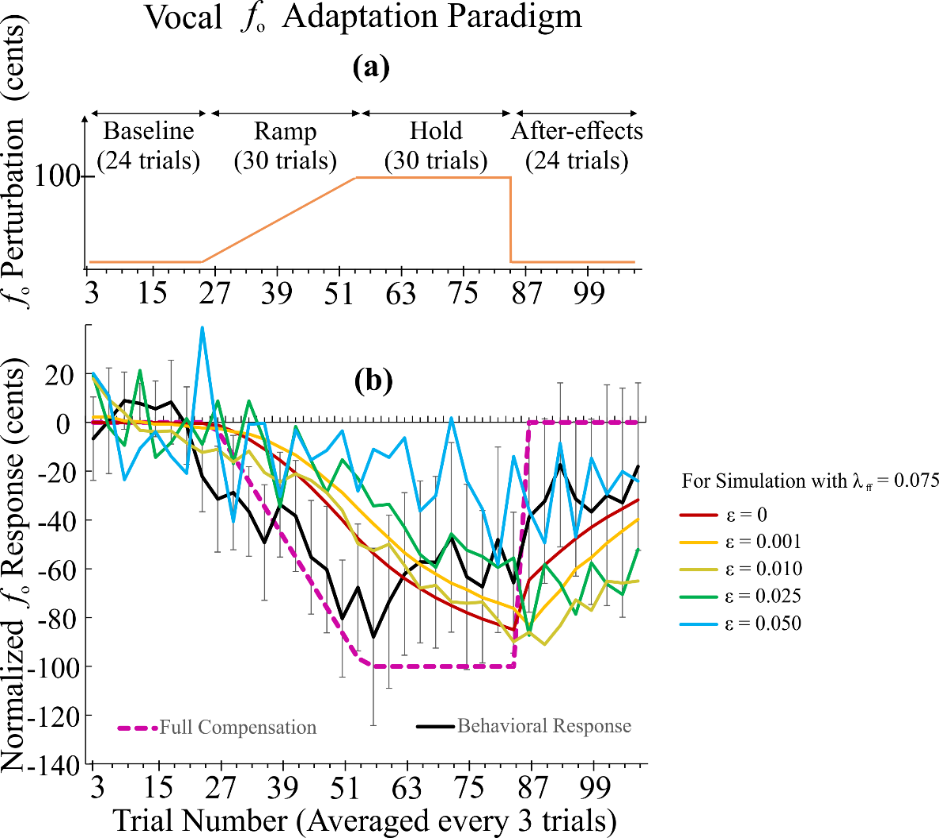
**

***Fig A: Simulation responses of LaDIVA for the vocal f_o_ adaptive paradigm with production noise compared with the mean group response (20 adults with typical speech) of the behavioral dataset.*** (a) Vocal *f_o_* perturbation across all 108 trials of the vocal *f_o_* adaptive paradigm. (b) Simulation responses for the vocal *f_o_* adaptation paradigm. All simulations in panel (b) were conducted under initial laryngeal muscle activation settings (Case B: a_CT_ = 0.169, a_TA_ = 0.175, and Ps = 800 Pa) and fixed control parameter settings (auditory feedback gain *g_audfb_* = 0.5, feedforward learning rate λ*_ff_* = 0.75). production noise parameter = ɛ. Group mean response of behavioral dataset in black with 95% CI error bars.

**References**

1. Cumbers, B. A. (2013). *Perceptual correlates of acoustic measures of vocal variability* The University of Wisconsin-Milwaukee].

2. Dhawale, A. K., Smith, M. A., & Ölveczky, B. P. (2017). The role of variability in motor learning. *Annual review of neuroscience, 40*, 479-498.

3. Scheerer, N. E., & Jones, J. A. (2012). The relationship between vocal accuracy and variability to the level of compensation to altered auditory feedback. *Neuroscience Letters, 529*(2), 128-132.
