# Supplementary material for: LaDIVA: A neurocomputational model providing laryngeal motor control for speech acquisition and production
Source: PLoS Comput Biol. 2022 Jun 23;18(6):e1010159. doi: 10.1371/journal.pcbi.1010159 (PMC9258861; doi:10.1371/journal.pcbi.1010159)
Supplement: Attachment — Submitted filename: PLOS_ResponsesToReviewerComments.docx [file pcbi.1010159.s004.docx]

Dear Editor and Reviewers,

Thank you for the time and effort taken for thoroughly reviewing the manuscript. We are very grateful for your comments and have addressed them to the best of our ability. We believe the quality of the article substantially improved with the feedback provided. Please see below for detailed responses and references to modifications we have done in the manuscript based on the reviewer comments.

Best regards,

Hasini Weerathunge
 **Authors’ Responses to Editor and Reviewer feedback**

**Editor:**

1. Your paper has now been reviewed by two experts in the field. They have both made extensive comments on your manuscript. Clearly the technical issues that were raised need to be addressed. Reviewer 2 has also raised important concerns on the scope of your model, more specifically that it does not address the principal deficits that are observed in voice disorders (phonation) and that it lacks a neurobiological component. Both of these were promised in the introduction. Optimally you could address these shortcomings by actually expanding your modeling efforts but, alternatively, by lowering expectations. You might be able to find the correct middle ground in your revised manuscript.

Authors: Thank you. We are very grateful for the feedback we have received regarding this modeling effort. We have addressed the technical issues that were raised, as well as the concerns regarding the scope of the model. In particular, we have added modifications to the manuscript to explain the modeling of neurobiological control of voice as well as a discussion of the principal deficits observed in voice disorders related to modeled components. We have also included additional descriptions of the models with regards to intrinsic delays and other information pertaining to technical queries raised by the reviewers.

**Reviewer #1:**

1. The comparisons between model and behavioral data remain qualitative. Because the authors claim this to be a validation study I recommend to present a more qualitative comparisons including statistics.

L273/4 “and the best fit for feedback gain was selected.” And also L292: The comparison between model and behavioral data is very qualitative. A simple difference between area under curve or curve-fit comparison would be stronger.

L293 I don’t see why lambda =0.1 has a better fit. A quantitative comparison would be stronger.

Authors: Thank you. We have added quantification in terms of goodness-of-fit statistics of R-squared, root mean squared error (RMSE), and absolute difference between area under the curves. After using graphical methods to estimate goodness-of-fit via visual interpretation (see figure 4 (c-d)), R-squared was calculated to estimate the percentage of variance of behavioral data explained by model simulations. The RMSE and absolute difference in area under the curves were calculated to estimate the accuracy of the model simulations compared to the behavioral dataset. We considered the simulations with the smallest absolute difference in the area under the curve as the best fit. See Page 17 Lines 298 – 300 and Table 2. See Page 18 Lines 329 – 331 and Table 3.

2. The experimental data to validate the BCM is mostly based on the Chhetri et al papers using graded muscle stimulation on the canine larynx. I suggest that the authors incorporate a discussion of these papers and also would like to point to recent papers of fluid-structure interaction models of the dog larynx from the Zheng lab (U of Maine, e.g. Geng et al JASA 2021 150,1176-1187). Their more accurate models could be included later in this laDiva as lumped parameter system

Authors: Thank you for the valuable feedback. We have included the graded muscle simulation citations to the manuscript to support the antagonistic activation between CT and TA muscles during phonation onset. The extended body cover model used in LaDIVA is based on an established low-order lumped-element model based on human data by Titze in 1984. The equations for the motion of vocal folds in the model were identical to the original paper by Story and Titze 1995, and model parameters were selected to simulate a male modal voice using muscle activation principles defined in Titze and Story 2002. We have modified the manuscript to clarify this information in the introduction section. We have modified the manuscript to clarify this information in the introduction section and added the higher order finite element model citations suggested by the reviewers in the manuscript. See Page 7 Lines 133 – 141. Based on the reviewer feedback, we have also included a discussion based on more accurate models that could be used in future work to increase the accuracy of vocal fold modeling of LaDIVA. The extended BCM was specially selected considering its simplicity as well as its scalability for future iterations of LaDIVA. For example, the triangular BCM is a more recent version of the extended BCM where independent and coordinated co-contraction of all agonist/antagonist intrinsic muscle pairs has been modeled. See Page 37 Lines 689 – 692.

3. The predicted muscle activity needs to be experimentally validated in follow-up studies. Modelling alone will never give an answer without validation. The authors should rephrase/town this down in their discussion.

Authors: We agree with the reviewer’s comment. In page 25 lines 413 - 417 in the discussion, we have mentioned existing EMG studies and how the resolution is too low/ noise is too high in EMG signals to capture EMG responses. We have rephrased this discussion and toned down the argument. See Page 25 Lines 421– 426.

4. The discussion is well balanced and includes current literature, but is very long and the paper would benefit from a more focused condensed discussion.

The discussion is very long and could be condensed

Authors: Thank you for your valuable comment. We have condensed the discussion, specially taking away redundant comparisons about simpleDIVA and LaDIVA models.

Specific comments
5. L108 these citations refer to EMG only

*Referred Text: The neural motor control and biomechanical mechanisms of typical and disordered laryngeal function involved in vocal production, and the interaction between them are poorly understood, partly due to the invasive nature of assessment and characterization of laryngeal function (31-36).*

Authors**:** Thank you. We have included citations for other invasive measurement techniques such as laryngeal electromyography techniques and/or contact pressure probes. See page 6 lines 116 – 117. See page 25 lines 413 – 417.

6. L130; can features be specified?

*Referred Text: Prior research has behaviorally characterized laryngeal motor control with externally imposed short-term (i.e. reflexive) and long-term (i.e. adaptive) alterations of auditory feedback of laryngeal features.*

Authors**:** We have modified the manuscript to specify the selected laryngeal features (i.e., vocal *f_o_*, vocal SPL, duration). See page 8 lines 148-149.

7. L166 physical quantity underlying the perceptual

*Referred Text: Vocal fo (acoustic correlate of pitch perception); vocal SPL (acoustic correlate of loudness perception)*

Authors: We have modified the definitions of vocal *f_o_* and vocal SPL to reflect the suggested change. I.e., vocal *f_o_* (physical quantity underlying the perceptual feature of pitch); vocal SPL (physical quantity underlying the perceptual feature of loudness).

See page 8 lines 149 – 150 and page 10 line 194.

8. L240 typo: missing citation 60

Authors: Added citation. See page 15 lines 268.

9. Fig 3a x-axis should be adjusted to same length as in C.

Authors: Modified. See Figure 3(a).

10. L301-302 The similar SPL along isolines of fo is surprising and may be a consequence of the simplicity of the vocal fold model driven at the same Ps.

Authors: We are thankful for the reviewer comments. However, this is the intended objective. We only wanted to change TA/CT activations to characterize the model in a controlled manner. Based on literature, we expected small SPL changes. To clarify this point we have modified the manuscript and added citations supporting small SPL changes for muscle activation changes in the range of 0.2. See page 19 - 20 lines 355 – 363. Moreover, we have plotted isolines of SPL in the CT/TA muscle activation plot for clarity. See Figure 4(b). Modifying subglottal pressure is part of the future work we will be carrying out in later iterations of the model. Thus, we have also modified the discussion section, future directions subsections to incorporate this information. See page 37 lines 683 – 689.

11. L307 the data only shows fo and not SPL. Could the authors also include the SPL results in Fig 4B?

Authors: Please note that the vocal SPL target range was maintained at 68 – 80 dB for the simulations to mimic the vocal SLP outputs expected at the behavioral study. As we maintained subglottal pressure constant and no acoustic coupling through all simulations, in the range of CT and TA muscle activations considered, the change in vocal SPL output was less than 1 dB SPL. Thus, the vocal SPL outputs did not exceed the target range, and no deviation in vocal SPL was observed for simulations. We have modified the manuscript to clarify this information better. See page 19 - 20 lines 356 – 363, and Figure 4(b) for manuscript modifications. The Limitations subsection under Discussion section in the manuscript lists the effects on vocal intensity when acoustic coupling is not considered. See page 32 lines 575 – 582.

12. L308 see also more recent simulations of these data from the Zheng lab (Geng et al).
*Referred Text: Prior literature suggests antagonistic relationships between vocal fo and aCT and aTA across the chest register (67).*

Authors: Thank you for the feedback. We have added the suggested citations in the manuscript. See Page 19 Line 357.

L315 This section would benefit from some additional explanation. I fail to see the major point. The model can clearly generate different pitch contours, but this is only a time-resolved implementation of what has been shown before. It is not clear how the prosodic contours were derived from the acoustic input signal.

Authors: Thank you for the constructive feedback. The capability of generating feedback controlled prosodic contours through LaDIVA model enables running speech pitch perturbation experiments to be simulated via the model. This has not been achieved before as vocal *f_o_* and vocal SPL were not controlled variables of DIVA model. We have included justification for these simulations in the manuscript in page 22 line 371 – 380.

The authors in the section above showed that the TA/CT space is redundant for fo and SPL. Thus, multiple activation levels can achieved the same acoustic output. At what positions did they start the simulations for Fig 4? L323 “simulated outputs fully overlapped with the original recording pitch contours.” The authors don’t include the evidence for this statement.

Authors: We have modified the manuscript under results section, prosodic contour simulations subsection based on reviewer comments to clarify the details. We have modified the prosodic contours in Figure 5 to separately showcase original acoustic signals and vocal *f_o_* traces as well as simulated *f_o_* contours. For simulations for Figure 5, Case B initial conditions for CT/TA activation levels were used. We have modified the manuscript to clarify this information. See page 22 - 23 lines 387 – 400.

13. I would say that the really interesting part is whether the resulting BCM related predicted physiological parameters, such as TA/CT activation, match experimental data.

L338-344 This is maybe true once there is substantial evidence that the predicted levels are correct: such evidence is currently lacking. The authors should tone down this section., but these EMG activation levels should be acquired to test the performance.
*Referred Text: The simulations also provided CT and TA laryngeal muscle activation levels across time for the paradigms, which are difficult to obtain in behavioral study settings. Only one previous study measured laryngeal electromyography responses to a vocal fo auditory feedback reflexive perturbation paradigm (33). However, the paradigms conducted in that study with conversational speech did not elicit observable patterns in CT and TA muscle activation levels and the authors speculated that the muscle activation levels were not detectable due to high levels of underlying neuromuscular signal noise. Thus, modeling efforts might be a more promising way forward in investigating intrinsic laryngeal muscle activity related to vocal fo reflexive paradigms.*

Authors: We agree with the reviewer comment. Thus, we have added further evidence provided via in vivo canine larynx experiments to support these observations and have additionally toned down the discussion, specifically mentioning that further research is required to confirm the simulated CT/TA muscle activation characteristics. See Page 25 lines 413 – 415, and lines 421 to 424.

**Reviewer #2:**

1. This manuscript presents a needed attempt to combine control models of speech production with a biologically realistic model of the vocal folds and larynx. Such a model has the potential to give insight into laryngeal control in typical speakers as well as help identify the mechanisms of vocal disfunction in various neurogenic voice disorders. However, I feel that the current manuscript has a problem with the scope of the work that has been done as it relates to these issues. I outline these in more detail, below. Additionally, there are several technical issues where the description of the model is unclear or incomplete. Lastly, there are some issues of interpretation or discussion of the results that I think are important to clarify.

Authors: Thank you for your comprehensive feedback, support, and interest in this study. We have done our best effort to respond to the technical issues, improve the focus, and clarify the points that you have raised.

Model scope:
2. I feel there the introduction and motivation for the work doesn’t match what is actually done. The introduction motivates the work primarily from the perspective of enabling a better understanding of voice disorders. However, the vast majority of vocal disorders (to my knowledge, and somewhat supported by looking at the references cited by the authors) are not problems with pitch control per se but structural or neurological problems resulting in phonation quality issues. This isn’t to say that this work isn’t useful—far from it—but the limitations of the current model to address the questions that are used to motivate it needs to be addressed.

Authors: Thank you for the constructive feedback. Vocal *f_o_* control is known to be affected in voice disorders and it is a key parameter in the voice analysis for these patients. However, we realize that the manuscript introduction may have not achieved the objective of fully summarizing atypical vocal fo control characteristics observed in behavioral studies carried out in individuals with hyperfunctional voice disorders. Thus, we have modified the manuscript to better clarify and summarize prior literature investigating atypical vocal *f_o_* production and control in individuals with Parkinson’s disease as well as individuals with hyperfunctional voice disorders. Page 8 lines 157 – 166. Modeling voice quality is part of the future work for the LaDIVA framework that is currently in progress. We have modified the manuscript to include this information under Discussion section, future directions subsection. Page 37 lines 689 – 692.

3. On a similar note, the motivation suggests that there is a need for a model that combines neural control of speech with a realistic phonation model. However, the current model makes no reference no neurobiologically grounded control. While this does not a priori limit the utility of the model, it does not speak to the need established by the authors in the introduction. Though the authors state that LaDIVA is a “neurocomputational model”, I don’t know that I agree—as presented, this seems like a pretty straightforward mathematical control model. That is not necessarily a problem in the abstract, but some discussion of the potential neurological basis of the model seems warranted given the authors stated intent.

Authors: The reviewer addresses a crucial point. We agree that the introduction of LaDIVA does not fully clarify the neurobiology of vocal motor control. However, the originating DIVA model is the most anatomically grounded neural network for speech motor control that has been developed up to date. DIVA model contains a set of mathematical equations representing the connectivity between different brain areas related to speech production. In a more granular level, DIVA model contains an artificial neural network with nodes representing neurons and synaptic weights representing connections strength between neurons. Using a neural network and connectivity based on neuronal clusters representing brain areas help DIVA model to clearly represent neurobiology of speech. DIVA model uses decades of behavioral and neuroimaging data to tie the components of the mathematical framework to neural substrates particularly related to articulation. DIVA model closely represents activations of brain regions for different control mechanisms of both voice and articulation based on these empirical findings. We have expanded the introduction to include DIVA model’s representation of the neurobiology of voice with this information. Given DIVA model has these attributes, they are inherited to LaDIVA model, as it is an extension retaining all characteristics of DIVA model. See page 5 lines 81 – 84 and lines 88 – 94.

4. I understand that the point of the current paper is to establish the model as a proof of concept, but I keep coming back to the motivations. Even with pitch control, some effort could be made to show the model may provide useful insights into vocal disorders. This could potentially be from some initial modeling of neurologically-based pitch control problems, or amplitude control problems (since that is also a feature of the model). Or perhaps showing that changes in some of the tunable control parameters could result in phonation quality changes, or really any demonstration that links a change in control to a (pathological) change in behavior. As it stands, the authors have shown the control architecture can control the laryngeal plant model, but not that this is a viable model of neurological impairment in vocal disorders. This is the critical need the authors establish, so some demonstration that the model may fill that need seems warranted.

Authors: We agree that phonation/voice quality is not a feature that is currently modeled in LaDIVA. Voice quality modifications require different outputs and voicing parameter (LCA/PCA muscle activations) to be controlled, which was out of the scope of this initial version of LaDIVA. Future implementations will consider additional inputs and outputs to extend the scope of the model. We have modified the manuscript to include this information under Discussion section, future directions subsection. Page 37 lines 689 – 692. That said, we emphasize that key pathological behavior can already be obtained with it. Auditory feedback gain and feedforward learning rate in LaDIVA are parameters that can be modified in this initial version to represent neurobiological impairments related to voice disorders. For example, prior studies suggest that persons with PD have hyperactive auditory feedback and weakened auditory-motor integration that causes impaired feedforward motor control. Thus, the neurological vocal motor control deficits in PD can be modeled using LaDIVA by increasing the auditory feedback gain and decreasing the feedforward learning rate. In the current study simulations, we have showcased how acoustic outcomes for reflexive and adaptive paradigms differ when auditory feedback gain and feedforward learning rate parameters are modified. Thereby we were able to showcase that reflexive and adaptive response magnitudes are reduced when auditory feedback gain and feedforward learning rate parameters are decreased, respectively.

More importantly, deficits in auditory motor integration has been identified via adaptive vocal fo studies conducted in individuals with vocal hyperfunction (Abur et al., 2021; Stepp et al., 2017). Thus, the suspected neurological vocal motor control deficits in vocal hyperfunction can be modeled using LaDIVA by decreasing the feedforward learning rate. However, vocal hyperfunction has not been sufficiently investigated to conclude if the origins are neurological, anatomical, or has components from both neural control and biomechanical control. The objective of LaDIVA is to combine a neurological model of vocal motor control and a biological model of peripheral laryngeal apparatus, thus providing a complex and comprehensive model that represents both neural and biomechanical control of voice. We can manipulate the model’s neurological as well as selected biomechanical parameters (which will grow in future versions) to investigate and gather insight on how the respective changes affects the vocal motor outcomes of the system. We have included this clarification in the manuscript under the discussion section in page 26 – 27 lines 427 – 449.

5. To some extent, the ability of the model to replicate compensatory and adaptive behavior is not particularly novel. This control architecture, which is what drives the changes in model behavior, has been shown to be capable of replicating these behaviors previously for auditory perturbations of vowel formants in supralarygneal articulatory control. The novel component here is largely the use of a new plant—the body cover model of the vocal folds—and the needed mapping (Jacobean) between acoustic and plant parameters. As long as that relationship is learnable, the model architecture is known to produce these results.

Authors: Thank you for your constructive feedback. We agree that LaDIVA control structure is an extension of the DIVA control structure where the vocal tract plant and control mechanisms are retained, and a new laryngeal plant and control mechanisms are added. However, this addition is indeed the novelty, as laryngeal motor control is not identical to articulatory motor control, as evidenced by behavioral observations in adults with typical speech function (Lester-Smith et al., 2020) as well as adults with neurogenic diseases (PD pathology differentially affecting laryngeal and articulatory motor control; hyperactive vocal *f_o_* feedback control e.g., Chen et al., 2013; Liu et al., 2012; Mollaei et al., 2016, 2019 and hypoactive vowel formant feedback control e.g.,Mollaei et al., 2016). The biomechanical constraints added via the laryngeal plant helps incorporate some of those differences into LaDIVA model in a physiologically relevant manner. For the current iteration we have used identical auditory feedback gain, and feedforward learning rates for both articulatory and laryngeal control. However, the model is scalable to explore their differences. Even with the current implementation, the model provides significant progress to better understand laryngeal motor control.

As outlined above, I think the demonstration of this established function would be substantially enhanced by examples of applications to voice disorders.

Authors: As we addressed in a previous point in reviewer feedback, auditory feedback gain and feedforward learning rate in LaDIVA model are parameters that can be modified to represent neurobiological impairments related to neurological (i.e., Parkinson’s disease) or functional voice disorders (i.e., vocal hyperfunction). We have showcased the changes in LaDIVA simulated acoustic outputs in vocal *f_o_* when auditory feedback gain and feedforward learning rate are modified. Thereby we were able to showcase that reflexive and adaptive response magnitudes are reduced when auditory feedback gain and feedforward learning rate parameters are decreased, respectively. Along with the neural control parameters, in future we hope to manipulate selected biomechanical parameters to investigate and gather insight on how the respective changes affects the vocal motor outcomes of the system. We have modified the discussion to clarify this information. See discussions section in page 26 lines 441 – 449.

Missing or incomplete technical information:
6. The adaptation component of the model is not described, as far as I can tell. In the traditional DIVA framework, adaptation to feedforward motor plans (motor trajectories) is accomplished by adding a time-shifted version of the feedback-driven compensatory motor commands to the existing motor trajectories, with a gain. No similar process is described in the current manuscript. Indeed, no process at all is described for updating the motor trajectories used by the feedforward control system.

Authors: We realize that that feedforward control component may have not been clearly described in the manuscript, so we have improved the way it is presented. Note that we initially provided minimal information about DIVA component due to existing literature on the model. However, we realize this is information in crucial for the general understanding of LaDIVA model as well. We have modified the manuscript to better explain the model’s adaptation component and its simulation implementations. See Materials and Methods section Feedforward controller subsection Equation 9 and Results section in Page 18 lines 317- 326.

7. The text often references a “feedforward gain” (gff). However, this parameter does not appear in the control diagrams of any of the equations. I believe that the feedforward gain is mislabelled as the learning rate (λff) in both the diagrams and equations, as this factor is simply a gain on the motor state error. For example, in equation 8 [FFmotor(x) = λff ∗ emotor(x)], where λff is multiplied by the error to generate the (current) feedforward motor command, that is then summed with the feedback commands in equation 9 to move the plant [Cmotor(x, i + 1) = FFmotor(x) + ∫(FBaud(x) + FBsomat(x)) dt] where C equals the “motor output”. So, this is really the feedforward gain and not the learning rate.

o Now, the process through which the feedforward trajectory (r) is updated maybe similar (and indeed actually rely on λff), but such an update is not specified in the methods.

Authors: Thank you for pointing this issue, which was an ambiguity on our end. We did refer to learning rate and feedforward gain to be similar. However, as original DIVA model does contain another feedforward gain parameter that we did not use, this warrants a clarification. We have updated the manuscript to clearly define feedforward gain and learning rate and specify that we used learning rate. The learning rate is selected as the parameter of interest because auditory motor integration is represented by learning rate. Feedback and feedforward weighting is represented by overall feedback gain and overall feedforward gain. See Material and Methods Section Feedforward controller subsection in Page 43 lines 831– 836, 838 – 840, and page 44 lines 845- 848. See page 44 equations 7, 7a – 7c, 8, and 9.

8. The simulation results in Figure 3 A show a latency of around 150 ms in the online compensatory response after the perturbation onset. This suggest that the model has built in delays in the auditory feedback processing pathway. However, no such delays are described in the methods.

Authors: Note that the original DIVA model has defined intrinsic delays to account for propagation delay between different brain areas based on prior literature. We agree that the delays in the neural control model are not clearly mentioned in the methods. We have modified the manuscript under methods section to incorporate a subsection on intrinsic delays. See Materials and Methods: Intrinsic Delays subsection in page 40 lines 765 – 791.

Moreover, we have modified all model equations and accompanying descriptions to reflect delayed components.

See page 42 equations 2, 3, and description at lines 805 - 806 and 807- 809.

See page 43 equations 5, 6, and description at lines 825 – 826 and 828 - 830.

See page 44 equations 7b, 8, and description at lines 860 – 866.

See page 45 equation 10, 10a, 10c, and description at lines 872 and 882 - 884.

See page 46 equation 11, 12, 2, and description at lines 902 - 906.

9. It’s not clear to me how the pitch and amplitude were determined at each time step. The description of acoustic simulation starts on line 763. My understanding here is that that the final acoustic output was synthesized at the end of the trial/utterance based on the history of trajectories generated by the controller. I am wondering how the acoustic signal was generated at each time step, however. For the Maeda plant, this is straightforward, as the plant position can be measured instantaneously (similar for the formants, which are estimated from the transfer function). I would assume that the BCM model takes some time to stabilize vocal production, however (this is suggested by the authors (line 288, where they say there is vocal onset variability up to 40ms in duration). So I am not clear on how the pitch and amplitude values were generated at each time step.

Authors: Thank you for the constructive feedback. The control loop in the DIVA implementation operates iteratively over 5 ms non-overlapping windows. At each 5 ms time step of an utterance, the motor representations are converted to auditory and somatosensory representations via the forward maps and error signals are calculated and inverse mapped to motor presentations. Thus, connecting DIVA and extended BCM together with a set of auditory task space parameters and motor mobility space parameters at their connection interface meant that simulations were required to be run every 5 ms across the two systems. Firstly, this limited the near real-time simulations of LaDIVA. Secondly, a 5 ms simulation in extended BCM was not sufficient to generate a glottal airflow signal stable enough to calculate a vocal *f*_o_ (i.e., due to vocal variability at the start of production in extended BCM). We agree that this process is not described properly such that readers understand that two different methods are used to generate 1) acoustic signal at each time step, vs 2) final acoustic output as a single production.

Thus, we have included clarifications in the Material and methods section of the manuscript. See Material and Methods, acoustic synthesizer subsection for a detailed explanation of the process carried out. Page 51 lines 1008 - 1015 and page 51 lines 1026 – 1031. Page 52 lines 1022– 1024. See Materials and Methods, generating forward maps subsection page 49 lines 956 - 965 for an explanation of the practical difficulties that caused the use of two methods.

Please note that the vocal onset variability of 40 ms mentioned in Results section under adaptive paradigm refers to the generic vocal variability in humans due to vocal fold adduction at vocal onset. It is not related to a model variability. We have modified the manuscript to remove this ambiguity. See page 18 line 324.

10. Line 438: “The JND for vocal fo in LaDIVA was set during all simulations to be significantly smaller compared to the maximum perturbation magnitude utilized in the perturbation paradigms”. There is no description of a JND setting in the methods/description of the model, or any type of perceptual thresholding.

Authors: We have added the JND thresholding information in the manuscript methods section as a subsection to better clarify JND. Materials and Methods: targets section page 40 lines 758 - 764 for more details.

11. Line 591:“five variables represent the vocal tract and are defined as constriction levels of six different places of constriction along the vocal tract (S1 − S6 ).” Is it five or six?

Authors: It is five places of constrictions along the vocal tract and the sixth place of constriction is the glottis, i.e, the voicing parameter. We have renamed it as glottal constriction to be consistent with the remainder of the manuscript. We have modified the manuscript to better clarify this point. See page 38 line 725

12. Line 601: “targets are saved as mobility space variables and converted to respective auditory and somatosensory task space reference targets when initially loaded for simulations.” This seems to be a pretty key element, but no description is given for how this “conversion” is accomplished. Some more detail is needed here.

Authors: We have added clarifications in the manuscript of how targets ae tuned in the Materials and Methods section under targets subsection. See page 39 lines 735 – 742.

13. Line 603: “As reference targets vary over time, they are defined as time series of minimum and maximum target values each domain variable can operate within (e.g., raudmin(y, t) and raudmax(y, t)). For the purpose of error calculation in feedback controllers, the midpoint between minimum and maximum values for each time point is considered as the target for each variable.” So, for all intents and purposes, the targets are point trajectories and not regions. I think this should be made explicit, as it is a departure from the standard implementation of DIVA. This is important, as it is my understanding that in the standard DIVA model (at least as typically described in published papers) errors are defined as the distance to the edge of the target region. So, this is a departure from previous work and may cause different behavior.

Authors: We agree with the reviewer. The original DIVA model also considers point trajectories and the same process is applied in LaDIVA model. We have corrected this information in the manuscript under Materials and Methods section targets subsection. See Page 39 lines 742 – 746.

14. Line 684: “The extended version of the BCM, including a revised glottal flow model with a posterior glottal gap introduced by Zañartu, Galindo (41), was also considered.” I don’t know what this means here—that the extended version was used? That is was not used? How did the results vary between the two versions of the model?

Authors: Thank you for the constructive feedback. The extended version of the BCM was the model used. It contains the original model components of Titze model and VF posturing component as an added feature. We have rephrased the sentence to clarify this point in the manuscript. See page 47 lines 910, 912 – 914.

15. Line 702: “Intrinsic laryngeal muscles also control VF posturing and tissue properties.” I assume that “intrinsic laryngeal muscles” means all the specified muscles (TA, CT, IA, LCA, PCA), but this should be made clear.

Authors: We have made modifications to the manuscript to clarify that in the current implementation, only TA and CT muscle activations were varied and IA, LCA, PCA remained fixed. In future iterations there are plans to incorporate a triangular BCM to independently control all 5 intrinsic muscle activations. See Materials and methods under subsection ‘Overview of extended BCM’ in page 48 lines 939 – 944.

16. Line 287: “This window was selected to target the feedforward response as the auditory feedback response is assumed to begin approximately 120 ms after perturbation onset”. The onset in the model is fully a function of the (unspecified) delays built in. So, rather than picking a time window based on human behavioral data, the authors should pick the time window of interest based on the latency response of the model. The window chosen seems like it may be fine for this given the simulations shown in Fig 3A, but I would clarify this point in the text (especially since trajectories for trials in the adaptation experiment are not shown—was the latency the same?).

Authors: The inbuilt delays in the model replicate behavioral data-based delays as close as possible. Thus, we believe the usage of behavioral analysis windows in the model is justifiable. However, this argument is not clearly stated in the manuscript. So, we added a separate section discussing the intrinsic delays defined in the model and how they are matched with empirical delays in the brain pathways. See intrinsic delays subsection under Materials and methods section in page 40 - 41 lines 765 - 791.

Interpretation of results:
17. I am not sure how informative the parameter setting exploration is for the auditory feedback gain in modeling the compensatory response. As I understand it, this is really and exploration of the RELATIVE gain between the feedforward controller and auditory feedback controller, rather than strictly an exploration of the auditory feedback gain, as presented. This is because, as the auditory feedback controller moves the system away from the specified motor trajectory, the feedforward controller will attempt to move back to that trajectory. So the results of a specific FB gain are only relevant for a fixed FF gain, which is fairly arbitrary. I think a more thorough exploration of these parameters is warranted. Or at a minimum, an acknowledgement that these results only hold for the fixed FF gain used.

Authors: We agree with the reviewer comment that this is a relative parameter exploration and we have pointed that out in the revised manuscript. However, note that prior research is consistent with the fixed feedforward gain approach as adults are observed to have fixed feedforward gain unless there is an anatomical change or sensory deficit that perturbs the system significantly. Even in these specific cases, the feedforward gain is not expected to differ across time spans relevant to behavioral paradigms. We have modified the manuscript to acknowledge that we used a fixed feedforward gain and the assumptions behind this selection. See Material and Methods, Feedforward controller subsection page 43 - 44 lines 840- 843.

In terms of the feedforward learning rate, we expected it to be constant for the duration of the reflexive paradigms as no persistent (i.e., sustained and predictable) errors were presented to the participants during reflexive paradigms that could cause feedforward learning. Reflexive paradigms contained sudden and unpredictable perturbations of auditory feedback that were designed to avoid adaptation effects. We have added clarifications of this information in the manuscript as per reviewer suggestion. See Results section page 17 lines 300 - 304.

18. Relatedly, what is the importance of a specific gain fitting the data well (i.e., that “We observed that gfb_aud = 0.7 provided an excellent fit for the behavioral mean vocal fo reflexive response”? Is it just that the model can fit the data? The argument for building the model was that it can help shed light on the neurobiologoical control of vocal pitch, so I would assume that such parameter fitting has some importance beyond just showing the model can fit the data.

Authors: We agree that the model is not initially intended for parameter fitting, as simpleDIVA is an ideal model for that. The implementation of LaDIVA is to provide a full and complex implementation of neural and biomechanical control of voice. Parameter fitting is an important feature of the neural control component, for which it was showcased here. For example, when exploring an individual with voice disorders, we can modify the BCM model to account for anatomical changes (e.g., Alzamendi et al., 2021, 2022), as we could adjust the gains in the neural model to represent atypical vocal motor control and analyze the changes in acoustic outcomes and auditory motor responses. This will help us investigate the causes of voice disorder based vocal features (vocal *f_o_* and SPL). We have incorporated clarifications to the manuscript regarding this point. See discussion section in page 30 lines 528 - 535.

19. There is a pretty big (and somewhat related) theoretical issue raised by the compensation results. Here, the compensatory response is only a fraction of the applied perturbation, even with an auditory feedback gain of 1 (it looks like the plateau of the response will be around 35 cents relative to the 100 cent perturbation). As the authors point out in the discussion (line 483), such partial compensation has often been attributed to competition between the auditory feedback system and the somatosensory feedback system. However, the current model does not include somatosensory feedback control for pitch and, as such, this result suggests that the partial compensation observed arises directly though competition between the feedback and feedforward control systems (perhaps in addition to competition with somatosensory feedback control). This sort of upends the typical story (and contradicts what the authors themselves write in the discussion), so I believe that some discussion is warranted.

Authors: Thank you for this important comment and observation. However, this is not a theoretical issue in the model. That said, we agree that it is not straightforward in the model overview how the compensation is calculated. Firstly, the model calculates vocal *f_o_* errors in Hz and the compensatory responses are presented in cents. As vocal *f_o_* is perceived logarithmically by humans and because participants have different baseline vocal *f_o_* values (i.e., male: 85 Hz – 155 Hz, female: 165 Hz – 255 Hz; Fitch & Holbrook, 1970), cents scale is used in behavioral studies to normalize auditory perturbations and responses in participants to provide perceivably similar perturbations. Secondly, as mentioned by reviewer earlier, error is calculated in LaDIVA similar to DIVA by taking the difference between the production and the edge of the target region. Thus, the error is calculated in Hz between the 100-cent perturbation and 5 cent target region maximum value. Thirdly, according to equation 9 (see below), the ‘equally weighted’ summation of the feedforward signal and feedback corrective error signal constitutes the model output. Therefore, even if 100% compensation is considered by setting auditory feedback gain = 1 and neglecting somatosensory feedback completely, only 50% of that total feedback signal gets carried over to the final output signal. We have included a calculation of how an auditory feedback gain of 1 (100% auditory feedback compensation) outputs a 50 cent vocal *f_o_* response in the supplemental material, as we believe this is an important clarification to make to the target audience of the paper. We thank the reviewer for raising this query and hope our response clarifies the query. See Supplemental material S3.

Please note that due to this comment we later found a calculation issue in the model simulations, where both auditory feedback gain parameter and overall feedback gain parameter was modified to generate the simulation responses in the initial manuscript that caused the acoustic output of vocal *f_o_* to plateau at 35 cents instead of 50 cents for auditory feedback gain = 1 condition. We are glad to have caught this error due to the reviewer’s comment and have redone all simulations in the manuscript to reflect this change. The supplemental figure S1 indicates a longer utterance length of 3 seconds for the vocal *f_o_* perturbation paradigm to indicate that the plateauing steady states at 50 cents although 50 cents is not achieved around 500 ms after perturbation onset (in Figure 3(c) the time series in only presented until 600 ms from perturbation onset).

$$\begin{aligned} C_{\mathrm{motor}}\left( x, i+1 \right)=\mathrm{FF}_{\mathrm{motor}}\left( x \right)+g_{\mathrm{fb}_{\mathrm{total}}}*\int\left( \mathrm{FB}_{\mathrm{aud}}\dot{(x)}+\mathrm{FB}_{\mathrm{somat}}\dot{(x)} \right) dt \#\left( 9 \right) \end{aligned}$$

20. There is a pretty substantial discussion comparing these results to the simpleDIVA model. I am not sure such a large discussion is warranted, as the models are radically different in both their architecture, and more importantly, intent. The current model is a control model of vocal and supralaryngeal production that is capable of learning. simpleDIVA is, conversely, a way to fit a set of equations to time-series data to estimate gain parameters and is NOT a control model.

Authors: We agree with the reviewer completely and thus have reduced the discussion section regarding comparisons to simpleDIVA.

21. Line 489: “This reasoning [the fact that there is no somatosensory feedback controller in the current model] may explain why LaDIVA’s adaptive simulations do not produce fo contours that fit empirical data as well as its reflexive simulations.” This is possible, but I’m not really convinced that this will solve the problem. Primarily because the largest difference between the simluations of adaptation and adaptation trajectories in real data are that the latter are noisy—they don’t monotonically increase. The difference between the real, noisy adaptation trajectories and the smooth adaptation trajectories produced by the model is clear in Fig 3B. Adding somatosensory feedback will not change this difference See, e.g., Villacorta, V. M., Perkell, J. S., & Guenther, F. H. (2007). Sensorimotor adaptation to feedback perturbations of vowel acoustics and its relation to perception. Journal of Acoustical Society of America, 122(4), 2306–2319. <https://doi.org/10.1121/1.2773966>, which uses the DIVA framework with somatosensory feedback control but still produces smooth adaptation trajectories. Adding this may change the overall shape of the trajectories to better match the data, which has a steeper rise and faster transition to a plateau, though again the simulations in Villacorta et al. show a similar shape to the simulations here, so it would seem that substantial changes in shape are unlikely. A similar difference in shape can be seen in the washout phase.

Authors: Thank you for the constructive feedback. We agree with the reviewer. Having no somatosensory feedback provides explanations as to why the adaptive responses reaches 100% compensation instead of the partial compensation observed in behavioral responses. However, it does not explain the variability seen across trials in the behavioral responses. One main reason for these noisy productions could be vocal variability. Vocal variability in production, perception, and learning are inherent characteristics in vocal productions and a main source of noisy productions. Adding noise-based variability at production, perception, and/or learning levels will be carried out in future iterations as it requires qualitative and quantitative specification of noise characteristics via behavioral paradigms. However, we have included generic levels of production noise to LaDIVA and observed the variations in adaptive paradigm responses and included our observations in Supplemental Material S4. We have modified the manuscript discussion section to include these clarifications. See page 27 - 28 lines 474 – 483.

More minor points:

22. Line 240: missing citation.

Authors: Added missing citation. See page 15 line 268.

23. Line 78: this is a citation for models of voice production, but citations neither citations 15 nor 16 treat voice production per se (they relate to the DIVA or GODIVA models of (supralaryngeal) control and planning).

Authors: Thank you for your valuable feedback. We have removed the noted citations. See page 3 line 76.

References

Abur, D., Subaciute, A., Kapsner-Smith, M., Segina, R. K., Tracy, L. F., Noordzij, J. P., & Stepp, C. E. (2021). Impaired auditory discrimination and auditory-motor integration in hyperfunctional voice disorders. *Scientific Reports, 11*(1), 1-11.

Alzamendi, G. A., Peterson, S. D., Erath, B. D., Hillman, R. E., & Zañartu, M. (2021). Triangular body-cover model of the vocal folds with coordinated activation of five intrinsic laryngeal muscles with applications to vocal hyperfunction. *arXiv preprint arXiv:2108.01115*.

Alzamendi, G. A., Peterson, S. D., Erath, B. D., Hillman, R. E., & Zañartu, M. (2022). Triangular body-cover model of the vocal folds with coordinated activation of the five intrinsic laryngeal muscles. *The Journal of the Acoustical Society of America, 151*(1), 17-30.

Chen, X., Zhu, X., Wang, E. Q., Chen, L., Li, W., Chen, Z., & Liu, H. (2013, Aug 21). Sensorimotor control of vocal pitch production in Parkinson's disease. *Brain Res, 1527*, 99-107. <https://doi.org/10.1016/j.brainres.2013.06.030>

Chhetri, D. K., Neubauer, J., & Berry, D. A. (2010). Graded activation of the intrinsic laryngeal muscles for vocal fold posturing. *The Journal of the Acoustical Society of America, 127*(4), EL127-EL133.

Chhetri, D. K., Neubauer, J., & Berry, D. A. (2012). Neuromuscular control of fundamental frequency and glottal posture at phonation onset. *The Journal of the Acoustical Society of America, 131*(2), 1401-1412.

Fitch, J. L., & Holbrook, A. (1970). Modal vocal fundamental frequency of young adults. *Archives of Otolaryngology, 92*(4), 379-382.

Lester-Smith, R. A., Daliri, A., Enos, N., Abur, D., Lupiani, A. A., Letcher, S., & Stepp, C. E. (2020). The Relation of Articulatory and Vocal Auditory–Motor Control in Typical Speakers. *Journal of Speech, Language, and Hearing Research, 63*(11), 3628-3642.

Liu, H., Wang, E. Q., Metman, L. V., & Larson, C. R. (2012). Vocal responses to perturbations in voice auditory feedback in individuals with Parkinson's disease. *PLoS One, 7*(3), e33629. <https://doi.org/10.1371/journal.pone.0033629>

Mollaei, F., Shiller, D. M., Baum, S. R., & Gracco, V. L. (2016, Sep 1). Sensorimotor control of vocal pitch and formant frequencies in Parkinson's disease. *Brain Res, 1646*, 269-277. <https://doi.org/10.1016/j.brainres.2016.06.013>

Mollaei, F., Shiller, D. M., Baum, S. R., & Gracco, V. L. (2019, Dec). The Relationship Between Speech Perceptual Discrimination and Speech Production in Parkinson's Disease. *Journal of Speech Language and Hearing Research, 62*(12), 4256-4268. <https://doi.org/10.1044/2019_Jslhr-S-18-0425>

Stepp, C. E., Lester-Smith, R. A., Abur, D., Daliri, A., Pieter Noordzij, J., & Lupiani, A. A. (2017). Evidence for auditory-motor impairment in individuals with hyperfunctional voice disorders. *Journal of Speech, Language, and Hearing Research, 60*(6), 1545-1550.
