# Supplementary material for: LaDIVA: A neurocomputational model providing laryngeal motor control for speech acquisition and production
Source: PLoS Comput Biol. 2022 Jun 23;18(6):e1010159. doi: 10.1371/journal.pcbi.1010159 (PMC9258861; doi:10.1371/journal.pcbi.1010159)
Supplement: Attachment — Submitted filename: ResponsesToReviewerFeedback_Revision2.docx [file pcbi.1010159.s005.docx]

**Author’s responses to Editor’s and Reviewer's Feedback**

Editor: Dear authors, could you also please publicly post the code for your model?

Author: The source code of LaDIVA model is currently situated at the publicly available GitHub repository at https://github.com/SteppLab/LaDIVA for reviewer reference. We are in the process of moving it to the website mentioned in the data availability statement in the following location (i.e., https://sites.bu.edu/guentherlab/software/diva-source-code/) with additional documentation at the moment.

Reviewer #2: I still think there’s perhaps a little too much emphasis but on the “neurobiological” aspect of the model. I do understand that the DIVA model uses a neural network implementation, but the examples of “neurobiological” parameters given in the paper are changes in feedback/learning gains—that is, tunable (and abstract!) mathematical parameters. That is, the authors aren’t suggesting that they can model changes in f0 responses in neurologoical disease through “lesions” to different components in the model or changes to the neural architecture, but by tuning the fixed model parameters (for example: “Thus, the suspected neurological vocal motor control deficits in vocal hyperfunction can be modeled using LaDIVA by decreasing the feedforward learning rate [line 440]”. That’s true, but it doesn’t give any insight into the neurobiology of WHY vocal hyperfunction results in this behavior—it’s simply a change in a tunable control parameter, and you could replicate this behavior in . Again, this isn’t a problem with the work itself—there’s a lot that can be learned from abstracted models! But it seems like there’s some overselling of what is actually happening here.

Author: We agree with the reviewer’s comment and modified the manuscript to reflect that the LaDIVA model’s capabilities to model vocal motor control remain in the neurocomputational domain. We have carefully removed language which might suggest that atypical neurobiology of different voice disorders can be specifically replicated in the model. We have also replaced the term “neurobiology” used with relation to the model with the term “neurocomputation” to represent the mathematically abstracted nature of the model. See the manuscript Discussion section at page 25: lines 435 – 436, and page 26: lines 453 – 455 for modifications.

I also still think that a discussion of the partial compensation seen in the model, even with the auditory feedback gain set to 1, is warranted. I don’t disagree with the authors reply at all—I think we are saying the same thing: namely, that since the corrective auditory feedback-driven motor command is (weighted and) summed with the feedforward command, that the model will maximally produce a partial 50% compensation. This is actually a strong prediction of the model: 1) that partial compensation is driven by competition between feedforward and feedback control systems, and not only between auditory and somatosensory feedback mechanisms, as is often stated in the literature and 2) that compensation should NEVER be above 50%, since this is the ceiling with a feedforward gain of 1.

Author: The reviewer points out an important idea. It seems that the confusion was in part caused by the incomplete explanation provided for the partial compensatory responses generated for vocal *f_o_* adaptation paradigms. Thus, we have modified the manuscript to better explain the reasons for partial compensatory responses for vocal *f_o_* adaptation paradigm. See Discussion section at page 33: lines 600 – 601, 604 – 606, and 609 – 612. We have supplemented this explanation with a discussion about the partial responses generated for vocal *f_o_* reflexive paradigms as suggested by the reviewer to clarify the model behavior further. See Discussion section at pages 33 – 34: lines 616 – 629.

L320: Relative to the analysis window (“In order to extract the acoustic response driven by feedforward motor commands alone, an analysis window from 40 ms to 120 ms after the vocal onset was selected. This analysis window was selected to target the feedforward motor command based response as auditory feedback is assumed to affect current vocal productions with a latency of 120 ms (63)”)—I still think the critical thing here is that you are choosing this window because you KNOW the model has not yet produced a compensatory response because you are controlling the delays, rather than being based on human behavioral data. E.g., if the model WERE producing a reflexive response in this window, it would not be a good window to assess feedforward learning IN THE MODEL, regardless of delays in real (human) data.

Author: We completely agree with the reviewer here. We have modified the manuscript to incorporate this clarification. See Results section at Page 18, Lines 324 – 329.

L376: I don’t follow what you mean by “We expect that this methodology can be used in the future to generate stimuli for auditory vocal fo perturbations of running speech.” If this is important, I would suggest explaining this in more detail.

Author: In dynamic running speech perturbations, different prosodic contours are generated by participants for running speech utterances and vocal *f_o_* perturbations are carried out at different stages of the utterance to investigate the task-relevance of compensatory responses to these perturbations. To model these dynamic perturbations, as an initial step, speech production models should be able to generate running speech utterances with different vocal *f_o_* contours. LaDIVA provides the capability to embed different prosodic contours to phrases as vocal *f_o_* is a controlled component. That is what we have demonstrated using examples in the simulations. We have incorporated this explanation in the manuscript under Results section at page 22, lines 376 – 381 and lines 384 – 386.
